# Silymarin as an Antioxidant Therapy in Chronic Liver Diseases: A Comprehensive Review

**DOI:** 10.7759/cureus.67083

**Published:** 2024-08-17

**Authors:** Devshree Dhande, Archana Dhok, Ashish Anjankar, Shailesh Nagpure

**Affiliations:** 1 Biochemistry, Jawaharlal Nehru Medical College, Datta Meghe Institute of Higher Education and Research, Wardha, IND; 2 Pharmacology, Dr. Rajendra Gode Medical College, Amravati, IND

**Keywords:** oxidative stress, non-alcoholic fatty liver disease (nafld), hepatoprotection, antioxidant therapy, chronic liver diseases, silymarin

## Abstract

Chronic liver diseases (CLDs) such as chronic hepatitis, cirrhosis, and non-alcoholic fatty liver disease (NAFLD) present significant global health challenges due to their high morbidity and mortality rates. Silymarin, a flavonoid complex derived from the seeds of the milk thistle plant (Silybum marianum), has been extensively studied for its hepatoprotective properties. This review aims to evaluate the role of silymarin as an antioxidant therapy in managing CLDs. We explore its efficacy, safety, and mechanisms of action through a comprehensive analysis of clinical trials and scientific studies.

Silymarin offers protective effects on the liver and shows promise in improving liver function and histological outcomes in various chronic liver conditions. Despite the promising results, further research is needed to fully elucidate the optimal dosing regimens, long-term safety, and potential drug interactions of silymarin. This review underscores the therapeutic potential of silymarin in CLDs and provides a foundation for future studies aimed at enhancing its clinical application.

## Introduction and background

Chronic liver diseases (CLDs) represent a diverse group of conditions characterized by persistent liver inflammation and damage, leading to significant morbidity and mortality worldwide [1]. Among these diseases, chronic hepatitis B and C, cirrhosis, and non-alcoholic fatty liver disease (NAFLD) are prominent. Chronic hepatitis B and C are viral infections that cause ongoing liver inflammation and can progress to cirrhosis and liver cancer [2]. Cirrhosis, often a consequence of various chronic liver insults, results in extensive fibrosis and compromised liver function. NAFLD, increasingly prevalent in the context of rising obesity rates, involves fat accumulation in liver cells without significant alcohol consumption and can progress to non-alcoholic steatohepatitis (NASH), cirrhosis, and liver failure [3].

Managing CLDs often involves addressing the underlying causes, managing symptoms, and preventing progression. Antioxidant therapies have gained attention due to their potential to mitigate oxidative stress, a key factor in liver damage and inflammation [4]. Oxidative stress arises from an imbalance between reactive oxygen species (ROS) and the liver’s antioxidant defenses, contributing to cellular injury and chronic inflammation. Antioxidant agents aim to neutralize ROS and restore cellular homeostasis, thereby slowing disease progression and improving liver function [5].

Silymarin is a complex flavonoid mixture derived from the seeds of the milk thistle plant (Silybum marianum). Traditionally used in herbal medicine, silymarin is well-regarded for its hepatoprotective properties. Its primary components include silybin (silybinin), silydianin, and silychristin, which collectively exhibit various pharmacological effects beneficial to liver health. Silybin, the most active component, is known for its potent antioxidant and anti-inflammatory properties [6]. The chemical composition of silymarin allows it to exert multiple protective effects on the liver. It enhances endogenous antioxidant defenses by increasing glutathione levels, a crucial intracellular antioxidant. Additionally, silymarin exhibits anti-inflammatory effects by modulating inflammatory pathways and reducing the production of pro-inflammatory cytokines. Its ability to stabilize cell membranes and promote hepatic regeneration further underscores its therapeutic potential in CLDs [7].

This review aims to comprehensively evaluate the role of silymarin as an antioxidant therapy in CLDs. The primary objectives are to assess silymarin's efficacy, safety, and mechanisms of action in this context. Firstly, the review seeks to evaluate the clinical evidence supporting the effectiveness of silymarin in improving liver function and managing symptoms in various CLDs, including NAFLD, chronic hepatitis, alcoholic liver disease (ALD), and cirrhosis. Secondly, it will review the safety profile of silymarin, including common adverse effects, potential interactions with other medications, and contraindications in specific patient populations. Finally, the review will investigate the underlying mechanisms by which silymarin exerts its hepatoprotective effects, focusing on its antioxidant, anti-inflammatory, and hepatoregenerative properties. By addressing these objectives, the review aims to provide a thorough understanding of silymarin’s therapeutic potential, guide clinicians in managing CLDs, and identify areas for future research.

## Review

Mechanisms of action of silymarin

Silymarin, the active component of milk thistle, exerts its beneficial effects through various mechanisms, contributing to its role as a therapeutic agent in CLDs. One primary mechanism is its antioxidant properties [8]. Silymarin is a potent scavenger of ROS, harmful byproducts of cellular metabolism that can lead to oxidative stress and cellular damage. By neutralizing these free radicals, silymarin helps protect liver cells from oxidative damage, thereby preserving liver function [9]. In addition to directly scavenging free radicals, silymarin enhances the activity of endogenous antioxidant enzymes, such as superoxide dismutase (SOD), catalase, and glutathione peroxidase (GPX). It also increases glutathione levels, a critical intracellular antioxidant vital in detoxifying harmful substances and maintaining cellular health [10].

Another significant mechanism of action of silymarin is its anti-inflammatory effects. Silymarin modulates various inflammatory pathways by inhibiting nuclear factor kappa B (NF-κB) activation. When activated, NF-κB is a transcription factor that leads to the expression of numerous pro-inflammatory genes [11]. By inhibiting this pathway, silymarin reduces the inflammatory response in the liver, which is crucial in conditions such as hepatitis and liver fibrosis. Furthermore, silymarin has been shown to decrease the levels of pro-inflammatory cytokines, including tumor necrosis factor-alpha (TNF-α) and interleukin-6 (IL-6). By reducing these cytokines, silymarin helps mitigate inflammation and its associated damage in liver tissues, promoting a healthier hepatic environment [9].

**Figure 1:**
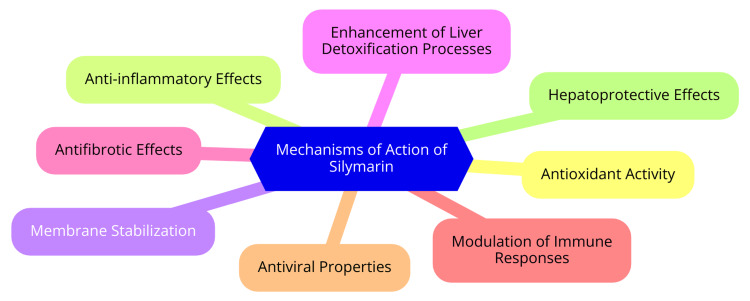
Silymarin - mechanisms of action Image Credit: Dr. Devshree Dhande

Silymarin also demonstrates hepatoprotective mechanisms that are vital for liver health. It inhibits the processes of liver fibrosis and cellular apoptosis (programmed cell death). This is achieved by blocking the activation of hepatic stellate cells, which are responsible for the development of fibrosis, and by promoting cell survival pathways. This dual action helps maintain liver architecture and function [8]. Additionally, clinical studies have shown that silymarin can restore elevated liver enzyme levels, such as alanine aminotransferase (ALT) and aspartate aminotransferase (AST), to normal ranges, indicating improved liver function. By protecting hepatocytes from damage and promoting their regeneration, silymarin supports the overall recovery and health of the liver [12]. Mechanisms of action of silymarin are shown in Figure 1.

Clinical evidence on silymarin in chronic liver diseases

Silymarin has been extensively studied for its potential benefits in treating NAFLD, a condition characterized by liver fat accumulation without significant alcohol consumption [13]. A systematic review and meta-analysis of eight randomized clinical trials found that silymarin significantly reduces liver enzyme levels, specifically ALT and AST, compared to placebo, even without concurrent weight loss. Notably, a trial involving 64 patients with NASH showed substantial reductions in ALT and AST levels after just eight weeks of treatment with 210 mg/day of silymarin [12].

In addition to lowering liver enzyme levels, silymarin has been associated with histological improvements in liver conditions related to NAFLD. A double-blind, placebo-controlled trial reported significant reductions in fibrosis scores among patients treated with 700 mg/day of silymarin over 48 weeks, suggesting a potential role in enhancing liver health and slowing NAFLD progression [14]. The impact of silymarin on chronic hepatitis B and C has also been investigated, particularly its effects on liver inflammation and function. While some studies suggest benefits in improving liver function tests, evidence regarding its direct influence on viral load remains inconclusive. Silymarin is often used alongside conventional antiviral treatments to complement their effects and improve overall liver health during treatment regimens [15-16]. Silymarin has shown beneficial effects on ALD as well. Clinical trials indicate that silymarin can improve liver function tests, reduce inflammation, and potentially mitigate fibrosis in ALD patients. For example, compared to placebo, silymarin-treated patients experienced significant biochemical improvements in liver function [17].

Research also supports silymarin's potential in managing cirrhosis and its complications. Evidence suggests it may slow disease progression by enhancing liver function and reducing oxidative stress. Some studies indicate benefits in managing cirrhotic complications like cirrhotic diabetes, highlighting its potential role in improving metabolic dysregulation [8]. Silymarin is generally safe and well-tolerated, even in patients with advanced liver disease. Clinical trials have reported no significant adverse effects, making it a viable long-term management option for cirrhotic patients. Overall, silymarin shows promise as a complementary therapy across various CLDs, particularly NAFLD and ALD, with evidence supporting its efficacy in improving liver enzymes and potentially aiding in histological improvements. However, further high-quality studies are needed to establish its precise role in managing chronic hepatitis and cirrhosis [18].

Safety and tolerability

Silymarin is generally considered safe for use in the management of CLDs. However, as with any therapeutic agent, it is crucial to be aware of potential adverse effects, contraindications, and drug interactions to make informed decisions regarding its use [4]. The most frequently reported adverse effects of silymarin are gastrointestinal symptoms. Some patients may experience nausea, particularly when the supplement is on an empty stomach. Diarrhea is also common, especially at higher doses, and mild abdominal discomfort or cramps can occur. While these symptoms are usually mild and self-limiting, they can affect patient compliance [19].

Allergic reactions to silymarin, although rare, have been documented. Patients may experience skin rashes, itching, or swelling, particularly those with known allergies to plants in the Asteraceae family, such as ragweed or marigolds. Additionally, silymarin may interact with liver enzymes involved in drug metabolism, which could alter the effects of certain medications [20]. Certain patient populations require special consideration when it comes to silymarin use. Pregnant and nursing women should approach its use with caution, as the safety of silymarin during pregnancy and lactation has not been well established. Similarly, there is limited safety data regarding its use in pediatric populations, so caution is advisable in children [21].

Patients with hormone-sensitive conditions, such as breast cancer or endometriosis, should use silymarin cautiously due to its phytoestrogenic properties. Furthermore, individuals with a history of severe allergies or hypersensitivity to silymarin or related compounds should avoid its use altogether [22]. Silymarin has the potential to interact with various medications, particularly those metabolized by cytochrome P450 enzymes. It may inhibit certain enzymes, such as CYP3A4 and CYP2C9, which can lead to altered metabolism of drugs processed by these pathways. This interaction could increase plasma concentrations of certain medications, enhancing their effects or side effects [23].

Additionally, silymarin may have a mild anticoagulant effect, raising concerns when used alongside anticoagulants like warfarin or antiplatelet medications such as aspirin, as it could increase the risk of bleeding. It is also important to note that silymarin has been shown to have hypoglycemic effects, which may enhance the effects of antidiabetic medications, potentially leading to hypoglycemia [24]. Caution is advised when combining silymarin with other herbal supplements that may affect liver function or interact with pharmacokinetics. A thorough patient history and medication review are essential to ensure silymarin's safe and effective use in clinical practice [9].

Dosage and administration

Silymarin is available in various dosage forms, including tablets, capsules, and liquid extracts. The recommended dosages can vary depending on the specific formulation and the condition being treated [25]. Tablet and capsule strengths typically range from 140 mg to 300 mg, often standardized to contain 70-80% silymarin. Liquid extracts, such as tinctures or standardized extracts, can have different concentrations, so adhering to the manufacturer’s guidelines is important. Some formulations may also offer silymarin as a powdered extract for mixing with food or beverages [26]. In clinical studies, typical dosages range from 140 to 600 mg daily, divided into two or three doses. Specific dosage recommendations are as follows: for ALD, 140 mg to 420 mg per day; for NAFLD, 420 mg to 700 mg per day, often divided into multiple doses; and for drug-induced liver injury (DILI), 140 mg to 600 mg per day, depending on the severity and clinical guidelines [27].

Silymarin can be taken with meals to enhance absorption and reduce gastrointestinal discomfort. However, some studies suggest that taking it on an empty stomach may improve bioavailability. It is important to know that silymarin may interact with certain medications, including anticoagulants and antidiabetic drugs. Patients should consult a healthcare provider before starting silymarin, especially if taking other medications [28]. The duration of treatment can vary significantly based on the specific liver condition and individual patient response. Clinical studies have frequently employed treatment durations ranging from 12 weeks to 6 months. Regular follow-up is advised to monitor liver function tests and evaluate the effectiveness of the treatment. Depending on clinical response and tolerability, dosage or treatment duration adjustments may be needed [29].

Comparison with other antioxidant therapies

Both efficacy and safety profiles are critical when comparing silymarin to vitamin E and other antioxidants. Silymarin has shown significant efficacy in reducing oxidative stress markers and improving liver function, often demonstrating superior effects to vitamin E in certain contexts. Research indicates that combining silymarin with vitamin E can significantly reduce malondialdehyde (MDA) levels, a marker of oxidative stress, simultaneously increasing GPX levels in patients undergoing hemodialysis. This suggests that the two agents may work synergistically to enhance antioxidant activity [30]. Regarding safety, both silymarin and vitamin E are generally well-tolerated, with a low incidence of adverse effects reported in clinical studies. Silymarin, in particular, is recognized for its excellent safety profile, making it a favorable choice for long-term use in managing CLDs. This is particularly important for patients requiring extended treatment regimens [31].

Cost-effectiveness is another essential factor in choosing antioxidant therapy. While comprehensive cost-effectiveness analyses are limited, silymarin is often considered an economically viable option due to its availability and relatively low cost compared to some pharmaceutical treatments. Additionally, patients may prefer silymarin because of its natural origin and the perceived lower risk of side effects than synthetic antioxidants like vitamin E. Combining silymarin with other treatments may also enhance patient adherence to therapy [32]. Silymarin has shown promising results when used with other hepatoprotective agents, which can amplify its therapeutic effects. For instance, studies have demonstrated that combining silymarin with vitamin E reduces oxidative stress markers more effectively than either agent alone and improves overall liver function in patients with conditions such as NAFLD and CLD.

This synergistic effect highlights the potential of incorporating silymarin into a multi-faceted treatment strategy [33]. Moreover, silymarin has been evaluated with other antioxidants, including vitamin C, coenzyme Q10, and selenomethionine. These combinations have improved liver enzyme levels and lipid profiles in patients with NAFLD, suggesting that their combined antioxidant capacity can offer enhanced hepatoprotection. The collaborative mechanisms of these agents may lead to more significant clinical benefits, making combination therapies an attractive option for managing CLDs [31].

Future directions and research needs

Despite the promising potential of silymarin in treating CLD, several gaps in current knowledge necessitate further investigation. One significant concern is the standardization of silymarin formulations. There is a lack of consensus on the optimal formulation, including the ideal dosage, treatment duration, and the specific disease stage for initiating therapy. Standardized formulations are crucial to ensure reliable and reproducible clinical outcomes, enabling healthcare providers to make informed treatment decisions [34]. Another critical area requiring attention is the long-term efficacy and safety of silymarin. While short-term studies have shown benefits, there is insufficient data on the long-term effects, particularly across diverse populations and varying stages of liver disease. Understanding these long-term implications is vital for establishing silymarin as a standard therapeutic option.

Additionally, further research is needed to clarify the precise mechanisms through which silymarin exerts antioxidant and hepatoprotective effects. Understanding these mechanisms will help optimize its therapeutic use and guide future research [35]. Robust clinical trials are also necessary to evaluate the effectiveness of silymarin in various liver conditions, such as NASH and DILI. Establishing clear guidelines for its use in these contexts will be essential for integrating silymarin into clinical practice [36]. Recent innovations in silymarin formulations and delivery methods are enhancing its therapeutic applications. Advances such as silymarin-Eurosil 85, which offer improved oral bioavailability, have shown potent antioxidant effects in preclinical models. These formulations may improve clinical outcomes by ensuring higher plasma concentrations of the active compound. This bioavailability improvement could significantly enhance silymarin's efficacy in treating liver diseases [28].

There is also growing interest in potential combination therapies that utilize silymarin alongside other therapeutic agents and lifestyle modifications. This synergistic approach may not only enhance patient compliance but also improve overall treatment efficacy for conditions like NAFLD and metabolic syndrome. Integrating silymarin into a comprehensive treatment plan could yield better patient outcomes [9]. Furthermore, emerging research suggests potential applications of silymarin in other liver-related conditions, such as chronic viral hepatitis and metabolic disorders associated with liver dysfunction. Exploring these applications could expand the therapeutic scope of silymarin, making it a versatile tool for managing liver health [9].

## Conclusions

Silymarin, a flavonoid complex derived from the milk thistle plant, has emerged as a promising antioxidant therapy for managing CLDs such as NAFLD, chronic hepatitis, ALD, and cirrhosis. Through its potent antioxidant, anti-inflammatory, and hepatoprotective properties, silymarin has shown efficacy in improving liver function, reducing oxidative stress, and mitigating liver inflammation. Clinical studies suggest that silymarin can effectively enhance endogenous antioxidant defenses, stabilize cell membranes, and promote hepatic regeneration, thereby contributing to the overall health and functionality of the liver. Despite its benefits, the safety profile of silymarin, including potential adverse effects and drug interactions, must be carefully considered, especially in specific patient populations. This comprehensive review underscores the therapeutic potential of silymarin in CLDs, providing valuable insights for clinicians and highlighting the need for further research to optimize its use and explore its full clinical benefits.
